# Proteome scale comparative modeling for conserved drug and vaccine targets identification in *Corynebacterium pseudotuberculosis*

**DOI:** 10.1186/1471-2164-15-S7-S3

**Published:** 2014-10-27

**Authors:** Syed Shah Hassan, Sandeep Tiwari, Luís Carlos Guimarães, Syed Babar Jamal, Edson Folador, Neha Barve Sharma, Siomar de Castro Soares, Síntia Almeida, Amjad Ali, Arshad Islam, Fabiana Dias Póvoa, Vinicius Augusto Carvalho de Abreu, Neha Jain, Antaripa Bhattacharya, Lucky Juneja, Anderson Miyoshi, Artur Silva, Debmalya Barh, Adrian Gustavo Turjanski, Vasco Azevedo, Rafaela Salgado Ferreira

**Affiliations:** 1 Laboratory of Cellular and Molecular Genetics, Department of General Biology, Federal University of Minas Gerais, Belo Horizonte, Minas Gerais, Brazil; 2 Departament of Biochemistry and Immunology, Federal University of Minas Gerais, Belo Horizonte, Minas Gerais, Brazil; 3 Institute of Biological Sciences, Federal University of Pará, Belém, Para, Brazil; 4 School of Biotechnology, Devi Ahilya University, Khandwa Road Campus, Indore, MP, India; 5 Centre for Genomics and Applied Gene Technology, Institute of Integrative Omics and Applied Biotechnology (IIOAB), Nonakuri, Purba Medinipur, West Bengal, India; 6 Department of Chemistry, Federal University of Minas Gerais, Belo Horizonte, Minas Gerais, Brazil; 7 Structural Bioinformatics Group, Institute of Physical Chemistry of Materials, Environment and Energy, University of Buenos Aires, Argentine

## Abstract

*Corynebacterium pseudotuberculosis* (Cp) is a pathogenic bacterium that causes caseous lymphadenitis (CLA), ulcerative lymphangitis, mastitis, and edematous to a broad spectrum of hosts, including ruminants, thereby threatening economic and dairy industries worldwide. Currently there is no effective drug or vaccine available against Cp. To identify new targets, we adopted a novel integrative strategy, which began with the prediction of the modelome (tridimensional protein structures for the proteome of an organism, generated through comparative modeling) for 15 previously sequenced *C. pseudotuberculosis* strains. This pan-modelomics approach identified a set of 331 conserved proteins having 95-100% intra-species sequence similarity. Next, we combined subtractive proteomics and modelomics to reveal a set of 10 Cp proteins, which may be essential for the bacteria. Of these, 4 proteins (tcsR, mtrA, nrdI, and ispH) were essential and non-host homologs (considering man, horse, cow and sheep as hosts) and satisfied all criteria of being putative targets. Additionally, we subjected these 4 proteins to virtual screening of a drug-like compound library. In all cases, molecules predicted to form favorable interactions and which showed high complementarity to the target were found among the top ranking compounds. The remaining 6 essential proteins (adk, gapA, glyA, fumC, gnd, and aspA) have homologs in the host proteomes. Their active site cavities were compared to the respective cavities in host proteins. We propose that some of these proteins can be selectively targeted using structure-based drug design approaches (SBDD). Our results facilitate the selection of *C. pseudotuberculosis* putative proteins for developing broad-spectrum novel drugs and vaccines. A few of the targets identified here have been validated in other microorganisms, suggesting that our modelome strategy is effective and can also be applicable to other pathogens.

## Background

Antimicrobial resistance involving a rapid loss of effectiveness in antibiotic treatment and the increasing number of multi-resistant microbial strains pose global challenges and threats. Thereby, efforts to find new drug and/or vaccine targets to control them are becoming indispensible. *Corynebacterium pseudotuberculosis *(Cp) is a pathogen of great veterinary and economic importance, since it affects animal livestock, mainly sheep and goats, worldwide, and its presence is reported in other mammals in several Arabic, Asiatic, East and West African and North and South American countries, as well as in Australia [1]. *C. pseudotuberculosis* is a Gram-positive, facultative intracellular, and pleomorphic organism; it is non-motile, although presenting fimbriae [2]. Based on *rpoB *gene (a β subunit of RNA polymerase), it shows a close phylogenetic relationship with other type strains of CMNR (*Corynebacterium, Mycobacterium, Nocardia *and *Rhodococcus*), a group that comprises genera of great medical, veterinary and biotechnological importance [1,3]. A recent study showed that phylogenetic analysis for the identification of *Corynebacterium *and other CMNR species based on *rpoB *gene sequences are more accurate than analyses based on 16S rRNA [4]. Its pathogenicity and biological impact have already led to the sequencing of various strains of this pathogen from a wide range of hosts [3]. The pathogen causes several infectious diseases in goat and sheep population (biovar *ovis*), including caseous lymphadenitis (CLA), a chronic contagious disease characterized by abscess formation in superficial lymph nodes and in subcutaneous tissues. In severe cases, biovar *equi *infects the lungs, kidneys, liver and spleen, thereby threatening the herd life of the infected animals [2,5]. The disease has been rarely reported in humans, as a result of occupational exposure, with symptoms similar to lymphadenitis abscesses [6-8]. The bacteria can survive for several weeks in soil in adverse conditions, what seems to contribute to its resistance and disease transmission [9,10]. Direct contact to infectious secretions or contaminated materials are the primary sources of pathogen transmission between animals, but most frequently the infection occurs through exposed skin lacerations [5]. Given the medical importance of Cp and a lack of efficient medicines, in this study we applied a computational strategy to search for new molecular targets from this bacterium.

Recently, computational approaches such as reverse vaccinology, differential genome analyses [11], subtractive and comparative microbial genomics have become popular for rapid identification of novel targets in the post genomic era [12], [13]. These approaches were used to identify targets in various human pathogens, like *Mycobacterium tuberculosis *[14], *Helicobacter pylori *[15], *Burkholderia pseudomalleii *[16], *Neisseria gonorrhea *[17], *Pseudomonas aeruginosa *[18] and *Salmonella typhi *[19]. In general, such approaches follow the principle that genes/proteins must be essential to the pathogen and preferably have no homology to the host proteins [20]. Nevertheless, essential targets that are homologous to their corresponding host proteins may also be molecular targets for structure-based selective inhibitors development. In this case, the targets must show significant differences in the active sites or in other druggable pockets, when pathogenic and host proteins are compared [21-23].

Once a molecular target is chosen, the conventional experimental methods for drug discovery consist of testing many synthetic molecules or natural products to identify lead compounds. Such practices are laborious, time consuming and require high investments [24,25]. On the other hand, computational methods for structure-based rational drug design can expedite the process of ligand identification and molecular understanding of interactions between receptor and ligand [26]. Such approaches are dependent on the availability of the structural information about the target protein. Considering the availability of experimental structures in PDB (Protein Data Bank) only for a low percentage of the known protein sequences, comparative modeling is frequently the method of choice for obtaining 3D coordinates for proteins of interest [27] for the development of specific drugs and docking analyses [28,29].

In this work, we used a modelomic approach for the predicted proteome of *C. pseudotuberculosis* species. This served to bridge the gap between raw genomic information and the identification of good therapeutic targets based on the three dimensional structures. The novelty of this strategy relies in using the structural information from high-throughput comparative modeling for large-scale proteomics data for inhibitor identification, potentially leading to the discovery of compounds able to prevent bacterial growth. The predicted proteomes of 15 *C. pseudotuberculosis* strains were modeled (pan-modelome) using the MHOLline workflow. Intra-species conserved proteome (core-modelome) with adequate 3D models was further filtered for their essential nature for the bacteria, using the database of essential genes (DEG). This led to the identification of 4 essential bacterial proteins without homologs in the host proteomes, which were employed in virtual screening of compound libraries. Furthermore, we investigated a set of 6 essential host homologs proteins. We observed residues of the predicted bacterial protein cavities that are completely different from the ones found in the homologous domains, and therefore could be specifically targeted. By applying this computational strategy we provide a final list of predicted putative targets in *C. pseudotuberculosis*, in biovar *ovis *and *equi*. They could provide an insight into designing of peptide vaccines, and identification of lead, natural and drug-like compounds that bind to these proteins.

## Materials and methods

### Genomes selection

Proteomes predicted based on the genomes of fifteen *C. pseudotuberculosis* strains, including both biovar *equi *and biovar *ovis *(Table 1**) **were used in this study. Most of these genomes were sequenced by our group and are available at NCBI. We downloaded the genome sequences in gbk format from the NCBI server (ftp://ftp.ncbi.nih.gov/genomes/Bacteria) and the corresponding protein sequences (curated CDSs) were exported using Artemis Annotation Tool [30] for further analyses.

**Table 1:** Strains of *C. pseudotuberculosis* employed in the pan-modelome study, and their respective information regarding genomes statistics, disease prevalence and broad-spectrum hosts.

Strains	GPID	NCBI Accession	Genome Size (Mb)	Number of Proteins	G+C%	Hosts/ Location	Nitrate's Reduction/ Biovar	Clinical Manifestation	Sequencing Technology
Cp1/06-A	73235	NC_017308.1	2.28	1,963	52.2	Horse/USA	Positive/*equi*	Abscess	Illumina
Cp31	73223	NC_017730.1	2.3	2,063	52.2	Buffalo/Egypt	Positive/*equi*	Abscess	Ion Torrent, SOLiD v3
Cp258	157069	NC_017945.1	2.31	2,088	52.1	Horse/Belgium	Positive/*equi*	Ulcerative lymphangitis	SOLiD v3
Cp316	71591	NC_016932.1	2.31	2,106	52.1	Horse/USA	Positive/*equi*	Abscess	Ion Torrent
CpCIP52.97	61117	NC_017307.1	2.32	2,057	52.1	Horse/Kenya	Positive/*equi*	Ulcerative Lymphangitis	SOLiD v2
Cp162	89445	NC_018019.1	2.29	2,002	52.2	Camel/UK	Positive/*equi*	Neck Abscess	SOLiD v3
CpP54B96	77871	NC_017031.1	2.34	2,084	52.2	Antelope/S. Africa	Negative/*ovis*	CLA Abscess	Ion Torrent, SOLiD v3
Cp267	73515	NC_017462.1	2.34	2,148	52.2	Lhama/USA	Negative/*ovis*	CLA Abscess	SOLiD v3
Cp1002	40687	NC_017300.1	2.34	2,097	52.2	Goat/Brazil	Negative/*ovis*	CLA Abscess	454, Sanger
Cp42/02-A	73233	NC_017306.1	2.34	2,051	52.2	Sheep/Australia	Negative/*ovis*	CLA Abscess	Illumina
CpC231	40875	NC_017301.1	2.33	2,095	52.2	Sheep/Australia	Negative/*ovis*	CLA Abscess	454, Sanger
CpI19	52845	NC_017303.1	2.34	2,099	52.2	Bovine/Israel	Negative/*ovis*	Bovine Mastitis Abscess	SOLiD v2
Cp3/99-5	73231	NC_016781.1	2.34	2,142	52.2	Sheep/Scotland	Negative/*ovis*	CLA	Illumina
CpPAT10	61115	NC_017305.1	2.34	2,089	52.2	Sheep/Argentina	Negative/*ovis*	Lung Abscess	SOLiD v2
CpFRC41	48979	NC_014329.1	2.34	2,104	52.2	Human/France	Negative/*ovis*	Necrotizing lymphadenitis	SOLiD v3

### Pan-modelome construction

A high throughput biological workflow, MHOLline (http://www.mholline.lncc.br), was used to predict the modelome (complete set of protein 3D models for the whole proteome) for each Cp strain. MHOLline uses the program MODELLER [31] for protein 3D structure prediction through comparative modeling. Furthermore, the workflow includes BLASTp (Basic Local Alignment Search Tool for Protein) [32], HMMTOP (Prediction of transmembrane helices and topology of proteins) [33], BATS (Blast Automatic Targeting for Structures), FILTERS, ECNGet (Get Enzyme Commission Number), MODELLER and PROCHECK [34] programs. The protocol used here was modified accordingly from the original work by Capriles et al., 2010 [35]. Briefly, the input files of protein sequences were used in FASTA format for all strains because the MHOLline accepts only .faa format files for the whole process. Firstly, MHOLline selected the template structures available at the Protein data Bank (PDB) via BLASTp (version 2.2.18), using the default parameters (e-value ≤ 10e^-5^). Secondly, the program BATS refined the BLASTp search for template sequence identification into different groups namely G0, G1, G2 and G3. Only the protein sequences in the group G2, which are characterized by an e-value ≤ 10e^-5^, Identity ≥ 0.25 and LVI ≤ 0.7 (where LVI is a length variation index of the BATS program for sequence coverage, the lower the LVI value, the higher the sequence coverage and vice versa) were selected. Among the MHOLline output files, the group G2 contained the largest number of protein sequences (≥ 50% for each input file). Subsequently, the "Filter" tool classified the group G2 sequences into seven distinct quality models groups, from "Very High" to "Very Low" depending on the quality of the template structure for a given query protein sequence. The program MODELLER then modeled all these groups in an automated manner. The number of sequences in the group G2 varies for each *C. pseudotuberculosis* strain. Only the first four distinct quality model groups of G2 were taken into consideration in this study, these were: 1- Very High quality model sequences (identity ≥ 75%) (LVI ≤ 0.1), 2- High quality model sequences (identity ≥ 50%) and < 75%) (LVI ≤ 0.1), 3- Good quality model sequences (identity ≥ 50%) (LVI > 0.1 and ≤ 0.3) and 4- Medium to Good quality models (identity ≥ 35% and < 50%) (LVI ≤ 0.3) (http://www.mholline.lncc.br). The percentage of identity represents identity between query and template sequences, a LVI ≤ 0.1 is equivalent to coverage of more than 90%, while LVI ≤ 0.3 corresponds to coverage of more than 70%. Therefore, all protein 3D models considered in this study were built from sequences for which there existed a template with identity ≥ 35% and LVI coverage over 70%. Later on, the ECNGet tool assigned an Enzyme Commission (EC) number to each sequence in G2, according to the best PDB template. The MODELLER (v9v5) program performed the automated global alignment and 3D protein model construction. Finally, the program PROCHECK (v3.5.4) evaluated the constructed models based on their stereo-chemical quality. Additionally, transmembrane regions in the input protein sequences were predicted by HMMTOP, for putative vaccine and drug targets identification.

### Identification of intra-species conserved genes/proteins

The words genes and proteins are interchangeably used here but they refer to the same protein target of the pathogen. For the identification of highly conserved proteins with 3D models in all Cp strains (≥ 95% sequence identity), the standalone release of NCBI BLASTp+ (v2.2.26) was acquired from the NCBI ftp site (ftp://ftp.ncbi.nlm.nih.gov/blast/executables/blast+/LATEST/), installed on a local machine and a search was performed for all strains using Cp1002 as a reference genome. The highly conserved proteins were selected using a comparative genomics/proteomics approach using an all-against-all BLASTp analysis with cut off values of *E = 0.0001 *[12,17,20,36].

### Analyses of essential and non-host homologous (ENH) proteins

To select conserved targets that were essential to the bacteria, a subtractive genomics approach was followed [20]. Briefly, the set of core-modelome proteins from *C. pseudotuberculosis* were subjected to the Database of Essential Genes (DEG) for homology analyses. DEG contains experimentally validated essential genes from 20 bacteria [37]. The BLASTp cutoff values used were: *E-value *= 0.0001, *bit score *≥100, *identity *≥ 35% [20].

Furthermore, the pool of essential genes was subjected to NCBI-BLASTp (*E-value = 0.0001, bit score ≥100, identity ≥ 35%*) against (human, equine, bovine and ovine proteomes) to identify essential non-host homologs targets [12]. The set of essential non-host homologous proteins were further crosschecked with the NCBI-BLASTp PDB database using default parameters to find any structural similarity with the available host homologs protein structures, keeping cutoff level to ≤ 15% for query coverage. These proteins were checked for their biochemical pathway using KEGG (Kyoto Encyclopedia of Genes and Genomes) [38], virulence using PAIDB (Pathogenicity island database) [39], functionality using UniProt (Universal Protein Resource) [40], and cellular localization using CELLO (subCELlular LOcalization predictor) [41]. The final list of targets was based on 12 criteria as described previously [20].

### Analyses of essential and host homologous (EH) proteins

We have extrapolated our analyses and also considered protein targets that were predicted as essential to bacterial survival but showed homology to host proteins. This was based on the possibility to find differences between bacterial and host proteins to rationally design inhibitors. The pool of essential protein targets that showed cut off values equal or higher than those for essential non-host homologs through NCBI-BLASTp was treated as host homologous proteins. These were also analyzed for pathway involvement, virulence, functional annotation and cellular localization like essential non-host homologous proteins. To verify the presence of significant residue differences in druggable protein cavities, a structural comparison was performed for each pathogen and their corresponding host protein through the molecular visualization program PyMOL (v1.5, Schrodinger, LLC) (http://www.pymol.org). The related published data of each template structure for each host homolog was also crosschecked for information about these residues, based on the PDB code of each template structure as input in the PDBelite server [42]. Catalytic Site Atlas (CSA) was also consulted to get robust information of the active site residues for the druggable enzyme targets [43]. CSA is a database documenting enzyme active sites and catalytic residues in enzymes of 3D structure and has 2 types of entry, original hand-annotated entries with literature references and homologous entries, found by PSI-BLAST alignment to an individual original entry, using an *e-value *cut-off of 0.00005. CSA can be accessed via a 4-letter PDB code. The equivalent residue that aligns in the query sequence to the catalytic residue found in the original entry is documented. Though the DoGSiteScorer predicts the druggable protein cavities, the host homologous proteins were further subjected to CASTp (Computed Atlas of Surface Topography of Proteins) [44], Pocket-Finder and Q-SiteFinder [45] to get more reliable and robust results about the druggable cavities of the target proteins.

### Prediction of druggable pockets

3D structure information and druggability analyses are important factors for prioritizing and validating putative pathogen targets [46,47]. As aforementioned, for druggability analyses, the final list of essential non-host and host homologous protein targets in PDB format, were subjected to DoGSiteScorer [48], an automated pocket detection and analysis tool for calculating the druggability of protein cavities. For each cavity detected the program returns the residues present in the pocket and a druggable score ranging from 0 to 1. The closer to 1 the obtained values are, the more druggable the protein cavity is predicted to be, i.e. the cavities are predicted to be more likely to bind ligands with high affinity [48]. The DoGSiteScorer also calculates volume, surface area, lipophilic surface, depth and other related parameters for each predicted cavity.

### Virtual screening and docking analyses

The ligand library was obtained from the ZINC database, containing 11,193 drug-like molecules, with Tanimoto cutoff level of 60% [49]. Proteins were inspected for structural errors such as missing atoms or erroneous bonds and protonation states in MVD (Molegro Virtual Docker) [50]. The cavities predicted with DogSiteScorer (druggability ≥ 0.80) for all protein targets, were compared with the cavities detected by MVD. The most druggable cavity, according to DogSiteScorer, was subjected to virtual screening. MVD includes three search algorithms for molecular docking namely MolDock Optimizer [50], MolDock Simplex Evolution (SE), and Iterated Simplex (IS). In this work the MolDock Optimizer search algorithm, which is based on a differential evolutionary algorithm, was employed. The default parameters used for the guided differential evolution algorithm are a) population size = 50, b) crossover rate = 0.9, and c) scaling factor = 0.5. The top ranked 200 compounds for each protein were analyzed in Chimera for shape complementarity and hydrogen bond interactions, leading to the selection of a final set of 10 compounds for each target protein.

## Results and discussion

### Modelome and common targets in *C. pseudotuberculosis* species

**Figure 1:**
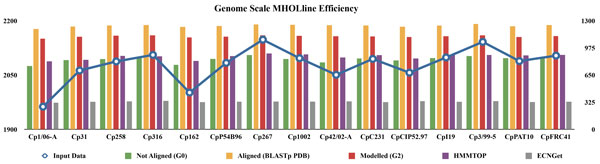
**High-throughputness (efficiency) of the MHOLline biological workflow for genome-scale modelome (3D models) prediction**. Predicted proteomes from the genomes of 15 *C. pseudotuberculosis* strains were fed to the MHOLline workflow in FASTA format. The blue line represents the number of input data, according to the left-hand side y-axis. The bars show the number in the form of MHOLline output data (according to the right-hand side y-axis) of: not aligned sequences (G0, green bars); sequences for which there is a template structure available at RCSB PDB (yellow bars); sequences with acceptable template structures that where modeled in the MHOLline workflow (G2, red bars); sequences with predicted transmembrane regions (HMMTOP, purple bars) and the number of sequences that were predicted as enzymes in each genome and were assigned an EC number (ECNGet, gray bars). The x-axis represents the *C. pseudotuberculosis* genomes used in this study.

**Figure 2:**
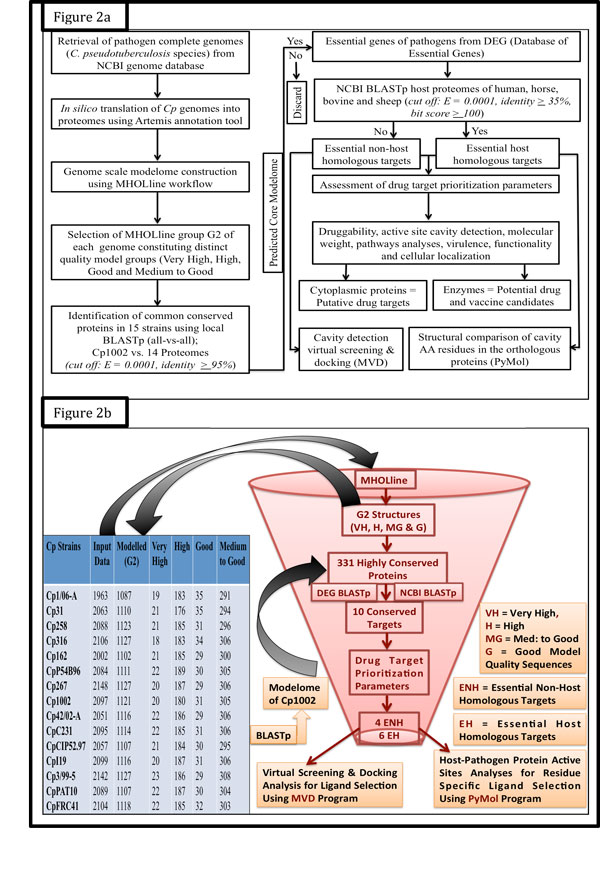
**Overview of different computational steps employed in the identification of putative essential targets (non-host homologous and host homologous) for drugs and vaccines from the core-proteome of 15 *C*. pseudotuberculosis strains**. **Figure 2b**. Intra-species subtractive modelomics workflow for conserved targets identification in *C*. pseudo tuberculosis species. The table (from left to right) represents the total number of protein sequences as an input data in fasta format fed to the MHOLline workflow (upper forward arrow). The remaining columns show the output data of group G2 (upper backward arrow), first by BATS and then by Filter tools of the MHOLline workflow respectively. Columns 4^th^-7^th ^constitute the number of protein sequences of different qualities of all 15 Cp strains, where the sequences of 14 Cp strains were compared using BLASTp, to the sequences of Cp1002 strain as reference, for the identification of conserved protein targets (core-modelome). The funnel shows how this workflow processes and filters a large quantity of genomic data for putative drug and vaccine targets identification of a pathogen.

Here we report the identification of common putative targets among 15 strains of *C. pseudotuberculosis* species based on the construction of genome scale protein three-dimensional structural models. Structural information of target proteins can aid in drug and/or vaccine design and in the discovery of new lead compounds [51]. The approach employed here generated high-confidence structural models through the MHOLline workflow **(**Figure 1**) **from orthologous protein. To identify the common conserved proteins with a sequence similarity of 95-100%, a comparative genomics approach was performed where all the BATS classified G2 sequences from "Very High" to "Medium to Good" quality, from 14 Cp strains, were aligned to the G2 sequences of Cp1002, assumed as a reference genome for this study. In total, a set of 331 protein sequences was selected, being conserved in all strains. An overview of the different steps involved in this computational approach for genome scale modelome and prioritization of putative drug and vaccine targets is given in Figure 2a-b.

### Identification of ENH and EH proteins as putative drug and/or vaccine targets

To identify essential proteins as putative therapeutic targets in *C. pseudotuberculosis*, from the set of core-modelome, these were compared to the Database of Essential Genes (DEG). Based on this filter, the number of selected targets was reduced drastically to a final set of only 10 targets. These were compared to the aforementioned corresponding host proteomes, leading to the identification of 4 essential non-host homologous proteins (ENH, Table 2) and 6 essential host homologous proteins (EH, Table 3).

**Table 2:** Drug and/or vaccine targets prioritization parameters and functional annotation of the four essential non-host homologous putative targets.

Gene and protein codes	Official full name	Number of cavities with Drug Score^a^ > 0.80	Number of cavities with Drug Score^a^ > 0.60 and < 0.80	Mol. Wt (KDa)^b^	Functions^c^	Cellular component^d^	Pathways^e^	Virulence^f^
Cp1002_0515 **MtrA**	DNA-binding response regulator mtrA	1	2	25.97	**MF: **DNA binding, two-component response regulator activity. **BP: **Intracellular signal transduction, regulation of transcription, DNA-dependent	Intracellular/ Cytoplasm	Two-component signaling systems	Yes

Cp1002_0742 **IspH**	4-hydroxy-3-methylbut-2-enyl diphosphatereductase	1	4	36.59	**MF: **Metal ion binding, 4-hydroxy-3-methylbut-2-en-1-yl diphosphate reductase activity, 3 iron, 4 sulfur cluster binding **EC: 1.17.1.2** **BP: **Dimethylallyldiphosphate biosynthetic process, isopentenyldiphosphate biosynthetic process, mevalonate-independent pathway	Cytoplasm	Inositol phosphate metabolism/ Pentose phosphate pathway/Terpene metabolism	Yes

Cp1002_1648 **TcsR**	Two-component system transcriptional regulatory protein	3	2	21.93	**MF: **Sequence-specific DNA binding, two-component response regulator activity, sequence-specific DNA binding transcription factor activity **BP: **Intracellular signal transduction, transcription, DNA-dependent	Intracellular/ Cytoplasm	Two-component system	Yes

Cp1002_1676 **Nrdl**	Ribonucleoside-diphosphatereductase alpha chain	1	1	88.02	**MF: **ATP binding, ribonucleoside-diphosphate reductase activity, thioredoxin disulfide as acceptor **BP: **DNA replication	Cytoplasm	Pyrimidine metabolism/ Purine metabolism	Yes

^a^Druggability predicted with DoGSiteScorer software. A druggability score above 0.60 is considered to be good, but a score above 0.80 is favored [48].

^b^Molecular weight was determined using ProtParam tool (http://web.expasy.org/protparam/).

^c^Molecular function (MF) and biological process (BP) for each target protein was determined using UniProt.

^d^Cellular localization of pathogen targets was performed using CELLO.

^e^KEGG was used to find the role of these targets in different cellular pathways.

^f^PAIDB was used to check if the putative targets are involved in pathogen's virulence.

**Table 3:** Drug and/or vaccine targets prioritization parameters and functional annotation of the six essential host homologous putative targets.

Gene and protein codes	Official full name	Number of cavities with Drug Score^a^ > 0.80	Number of cavities with Drug Score^a^ > 0.60 and < 0.80	Mol. Wt (KDa)^b^	Functions^c^	Cellular component^d^	Pathways^e^	Virulence^f^
Cp1002_0385 **Adk**	Adenylate kinase	**1**	0	24.120	**MF: **Kinase, Transferase, ATP binding **BP: **Nucleotide biosynthesis **EC 2.7.4.3**	Cytoplasm	Purine metabolism; AMP biosynthesis via salvage pathway	**Yes**

Cp1002_0692 **GapA**	Glyceraldehyde-3-phosphate dehydrogenase A	**2**	1	51.918	**MF: **Oxidoreductase, NAD binding, NADP binding, **BP: **glucose metabolic process **EC 1.2.1.13**	Cytoplasm	Glycolysis/Gluconeogenesis	**Yes**

Cp1002_0728 **GlyA**	Serine hydroxymethyltransferase	**2**	1	46.187	**MF: **Methyltransferase, Transferase **BP: **Amino-acid biosynthesis One-carbon metabolism **EC 2.1.2.1**	Cytoplasm	Amino-acid biosynthesis; glycine biosynthesis; One-carbon metabolism; tetrahydrofolate interconversion.	**Yes**

Cp1002_0738 **FumC**	Fumaratehydratase class II	**2**	0	49.767	**MF: **Lyase **BP: **Tricarboxylic acid cycle **EC 4.2.1.2**	Cytoplasm	Carbohydrate metabolism; tricarboxylic acid cycle; (S)-malate from fumarate	**Yes**

Cp1002_1005 **Gnd**	6-phosphogluconate dehydrogenase	**3**	5	53.669	**MF: **Oxidoreductase **BP: **Pentose shunt **EC 1.1.1.44**	Cytoplasm	Carbohydrate degradation; pentose phosphate pathway;	**No**

Cp1002_1042 **AspA**	Aspartate ammonia-lyase	**2**	4	52.277	**MF: Lyase** **EC 4.3.1.1**	Cytoplasm	Alanine, aspartate and glutamate metabolism, Nitrogen metabolism	**Yes**

^a^Druggability predicted with DoGSiteScorer software. A druggability score above 0.60 is usually considered, but a score above 0.80 is favored [48].

^b^Molecular weight was determined using ProtParam tool (http://web.expasy.org/protparam/).

^c^Molecular function (MF) and biological process (BP) for each target protein was determined using UniProt.

^d^Cellular localization of pathogen targets was performed using CELLO.

^e^KEGG was used to find the role of these targets in different cellular pathways.

^f^PAIDB was used to check if the putative targets are involved in pathogen's virulence.

Among the ENH proteins, two targets were selected from a bacterial unique pathway, the two component signaling system. These targets are tcsR (two-component response regulator) and mtrA (two component sensory transduction transcriptional regulatory protein). While the tcsR is a novel protein target, as it is has not been described so far as a target in any organism, mtrA has been already reported as a target in *Mycobacterium *[52] and provides multidrug resistance to *Mycobacterium avium *[53]. Therefore, targeting mtrA in *C. pseudotuberculosis* may also be effective in controlling the infection of CLA. The remaining ENH protein targets, nrdI and ispH, also participate in biochemical pathways. NrdI (ribonucleoside-diphosphate reductase alpha chain) is a flavodoxin which contains a diferric-tyrosyl radical cofactor and it is involved in nucleotide metabolism in *E. coli *[54]. It has been reported as a putative target in several pathogens including *C. pseudotuberculosis, Corynebacterium diphtheriae* and *Mycobacterium tuberculosis *[20]. The target ispH (4-hydroxy-3-methylbut-2-enyl diphosphate reductase; EC 1.17.1.2) is an essential cytoplasmic enzyme in *Escherichia coli *[55]. This iron-sulfur protein plays a crucial role in terpene metabolism of various pathogenic bacteria [56,57] and it is a predicted target in *Salmonella tyhpimurium *[58] and *Plasmodium falciparum *[59]. It should be noted that according to the cut off threshold for NCBI-BLASTp that we have followed, ispH shows homology only to the human host. So, if human is not considered as a possible host, ispH can also be considered as a common putative target. The roles of these proteins in different metabolic pathways was confirmed from KEGG [38] and METACYC [60] databases.

### Prioritization parameters of drug and/or vaccine targets

Previous studies have shown several factors that can aid in determining the suitability of therapeutic targets [46]. The availability of 3D structural information, the main approach of our study, is very helpful in drug development. Other important factors for drug targets include preferred low MW and high druggability. On the other hand, for vaccine targets the information about subcellular localization is important and proteins that contain transmembrane motifs are preferred [36,46,61,62]. We have determined most of these prioritizing properties for the 10 essential proteins (Table 2 &3). Interestingly, according to the target-prioritizing criterion, all targets have a low MW, and are predicted to be localized in the cytoplasmic compartment of the Cp. Druggability evaluation with DoGSiteScorer [48] for all conserved targets allowed the prediction of numerous druggable cavities with at least one druggable cavity for each Cp target. For the 4 ENH proteins tcsR, mtrA, nrdI, and ispH, 3, 5, 5 and 2 cavities with score ≥ 0.80 were observed respectively. For each protein, the cavity that exhibited the highest druggability score was selected for docking analyses. For 6 EH targets, adk, gapA, glyA, fumC, gnd, and aspA, 1, 3, 3, 2, 8 and 6 cavities were observed respectively according to the aforementioned druggability score criteria (Table 2 &3). Here, in each case, the most druggable predicted cavity was structurally compared with the cavities in respective host proteins.

### Virtual screening and molecular docking analyses of ENH targets

For each ENH target protein (mtrA, ispH, tcsR and nrdl), the top 200 drug-like molecules from virtual screening were visually inspected to select 10 molecules that showed favorable interactions with the target. The biological importance of each target and an analysis of the predicted protein-ligand interaction are described below. ZINC codes and MolDock scores of selected ligands, the number of hydrogen bonds as well as protein residues involved in these interactions, are shown in a table for each target protein (Tables 4, 5, 6, 7. Figures showing the predicted binding mode for one of the 10 selected ligands are also shown for each target (Additional files 1, 2, 3, 4, 5).

**Table 4:** ZINC codes, MolDock scores and predicted hydrogen bonds for the ten compounds selected among the top ranking 200 molecules against **Cp1002_0515 **(**MtrA**, DNA-binding response regulator).

ZINC IDs	MolDock score	Number of H-bonds/ residues interacting with the compound
75109074	-130.402	3 Thr73, Asp48, Arg116

12117405	-115.838	3 Arg119, Arg118, Ala115

02546720	-113.761	3 Thr73, Arg119

40266587	-116.119	2 Asp48, Leu117

71405274	-113.264	2 Arg116, Asp97

05687366	-111.376	2 Arg119, Asp48

04730243	-109.609	2 Arg119, Asp157

19720976	-109.061	2 Arg119

72342680	-108.299	2 Arg119, Asp157

**Table 5:** ZINC codes, MolDock scores and predicted hydrogen bonds for the ten compounds selected among the top ranking 200 molecules against **Cp1002_0742 **(**IspH**, 4-hydroxy-3-methyl but-2-enyl diphosphate reductase).

ZINC IDs	MolDock score	Number of H-bonds/ residues interacting with the compound
00510419	-151.376	7 Cys39, His68, Thr225, Ser250, Asn252

00529019	-129.348	5 His68, Ser250, Asn252, Thr193, Thr193

04344036	-135.156	8 Thr193, His151, His68, Ser251, Asn252, Ser250, Asn252

04632419	-136.984	6 Cys39, Gly41, Ala100, Cys222, Thr193, Asn252

04730243	-129.414	10 Cys222, Thr193, Asn252, His151, Ser250, His68, Ser250

05479451	-129.963	9 Asn252, Ser250, Ser251, His68, Cys123, Cys39, Gly41

05775454	-161.806	3 Asn252, Thr193, His68

16941408	-126.163	6 Thr193, Asn252, Asn252, Ser250

04622741	-127.816	12 Cys39, Cys123, His68, Cys222, Ser250, Ser251, His151, Thr193

14017317	-129.664	8 Cys39, Glu153, His68, Asn252, Ser251, Asn252, His151, Thr193

**Table 6:** ZINC codes, MolDock scores and predicted hydrogen bonds for the ten compounds selected among the top ranking 200 molecules against **Cp1002_1648 **(**TcsR,** Two component transcriptional regulator).

ZINC IDs	MolDock score	Number of H-bonds/ residues interacting with the compound
00510419	-167.633	3 Val76, Gln185, Asn193

01617096	-146.178	3 Ala74, Gln185, Arg191

32911447	-148.424	3 Gln185, Ala70, Arg193

00091802	-143.287	3 Val76, Ala51

67847806	-156.655	4 Thr75, Thr75, Pro48, Val76

19399766	-160.743	3 Val76, Val76, Ala51

16980834	-147.631	4 Ala66, Val76

06269029	-145.277	4 Thr75, Pro48, Val76

05934077	-145.785	3 Arg191, Gln185

01647971	-167.152	3 Thr75, Val76, Ala51

**Table 7:** ZINC codes, MolDock scores and predicted hydrogen bonds for the ten compounds selected among the top ranking 200 molecules against **Cp1002_1676 **(**NrdI**).

ZINC IDs	MolDock score	Number of H-bonds/ residues interacting with the compound
01585114	-151.406	6 Ser8, Ser7, Thr13, Asn12

04721321	-144.134	7 Ser8, Ser7, Thr13, Leu116

17023683	-140.718	6 Ser7, Ser8, Thr13, Thr13, Thr13

00510419	-154.064	4 Thr10, Thr13, Ser8

01417445	-138.997	4 Thr13, Tyr49, Ser8, Ser7

00042420	-135.363	6 Tyr49, Ser7, Ser8, Thr13, Thr13, Thr13

00408361	-133.535	6 Thr13, Ser7, Ser8, Tyr49, Thr48

15830653	-153.83	4 Ser7, Thr13, Tyr49

00032839	-139.327	6 Ser8, Ser7, Thr13, Thr13, Thr13

48212336	-137.675	6 Ser7, Ser8, Ser8, Thr13, Ser54

**Cp1002_0515 **(**MtrA**, DNA-binding response regulator) is part of the two-component signal transduction system consisting of the sensor kinase (Histidine protein kinases, HKs) and the response regulator, MtrB and MtrA respectively. This system is highly conserved in *Corynebacteria *and *Mycobacteria *and it is essential for their survival to adapt to environmental changes. Homologs of MtrA and MtrB are present in many species of the genera *Corynebacterium, Mycobacterium, Nocardia, Rhodococcus *(CMNR), and others like *Thermomonospora, Leifsonia, Streptomyces, Propionibacterium*, and *Bifidobacterium *[63]. MtrA represents the fourth family member of the OmpR/PhoB family of response regulators. Like other family members, MtrA has been reported to be essential in *M. tuberculosis *[64]. It possesses an N-terminal regulatory domain and a C-terminal helix-turn-helix DNA-binding domain, already indicating that this response regulator functions as a transcriptional regulator, with phosphorylation of the regulatory domain modulating the activity of the protein [65]. Based on a comparison with a crystallographic structure of the MtrA template (2GWR, MtrA from *M. tuberculosis*), the active site residues involved in H-bond interactions with the crystallographic ligand are Val145, Gln151, Ile152 and Leu154. Although none of these residues is predicted to form hydrogen bonds with the ten selected docked ligands, these molecules were predicted to interact with other residues in the pocket. Table 4 shows the 10 selected ligands according to their minimum energy values and number of hydrogen bond interactions. **ZINC75109074 **(N-benzyl-N-[[2-(2-thienyl)-1H-imidazol-4-yl] methyl] prop-2-en-1-amine) is shown here as the top scoring ligand **(**Additional file 1**)**.

**Cp1002_0742 **(**IspH**, 4-hydroxy-3-methylbut-2-enyl diphosphate reductase) is an iron-sulfur oxidoreductase enzyme that plays a key role in the metabolism of terpenes in several pathogens. Terpenes constitute a large class of natural compounds. Their biosynthesis initiates with the building blocks isopentenyl-diphosphate (IPP) and dimethylallyldiphosphate (DMAPP), and differs in bacteria and mammals [57]. In bacteria and other pathogenic microorganisms the enzyme IspH catalyzes the last step in the production of IPP and DMAPP. The three structural units of the enzyme harbor a cubic iron-sulfur cluster at their center, enabling the enzyme to accomplish a challenging reaction by converting an allyl alcohol to two isoprene components. The iron-sulfur proteins normally participate in electron transfers. The IspH enzyme, thereby, in a similar fashion, binds the substrate directly to the iron-sulfur cluster [57]. In the template crystal structure of IspH (PDB 3KE8), it has been shown that His41, His74, His124, Thr167, Ser225, Ser226, Asn227 and Ser269 are the active site residues that are involved in hydrogen bond interactions with the ligand 4-hydroxy-3-methylbutyldiphosphate (EIP). Also, Cys12, Cys96, Cys197 and EIP have been shown to make metal interaction with the Fe_4_S_4 _(Iron/Sulfur Cluster). Although the ten selected drug-like compounds (Table 5**) **did not show any interaction with the aforementioned IspH residues, they are predicted to make very good hydrogen bond interactions with other surrounding residues of the predicted cavity. The predicted binding mode of the best scoring compound, **ZINC00510419 **is shown in Additional file 2. Good shape complementarity and 6 hydrogen bond interactions are observed in this complex.

**Cp1002_1648 **(**TcsR, **Two component transcriptional regulator) is a novel target without host homologs proteins. Differently from MtrA and IspH, in this case the template structure from *Escherichia coli *for TcsR did not contain any ligand (PDB 1A04), and no reported information was found about the ligand-residues interactions in their cavities. Therefore, among the cavities identified by MVD, the best cavity for virtual screening analysis was simply chosen based on the highest druggability score by the DogSiteScorer. Compound **ZINC00510419 **(Additional file 3**) **was the top-ranking compound, forming a network of 3 hydrogen bonds with Val76, Gln185 and Asn193. Table 6 lists the 10 compounds selected for this target.

**Cp1002_1676 **(**NrdI, **protein) belongs to the nrdI protein family, a unique group of metalloenzymes that are essential for cell-proliferation [66]. It is classified as a ribonucleotide reductase (RNR), an iron-dependent enzyme that belongs to class Oxidoreductases (EC 1.17.4.1) acting on CH or CH_2 _groups with a disulfide as acceptor [67]. The class Ia enzyme supplies deoxynucleotides during normal aerobic growth. The class Ib RNR plays a similar role although its function in *E. coli *is not clear, but it is reported to be expressed under oxidative stress and iron-limited conditions [68]. Class I RNR enzymes have two homodimeric subunits, α2 (NrdE), where nucleotide reduction takes place, and β2 (NrdF) containing an unidentified metallocofactor for initiating nucleotide reduction in α2. Although the exact function of NrdI within RNR has not yet been fully characterized, it is found in the same operon as NrdE and NrdF, and encodes an unusual flavodoxin, a bacterial electron-transfer protein that includes a flavin mononucleotide that has been proposed to be involved in metallocofactor biosynthesis and/or maintenance. It has also been proposed that NrdI plays an important role in *E. coli *class Ib RNR cluster assembly. Recent *in vitro *studies have shown that a stable diferric-tyrosyl radical (FeIII2-Y·) and dimanganese (III)-Y· (MnIII2-Y·) cofactors are active in nucleotide reduction [69]. The first one can be formed by self-assembly from FeII and O_2 _while the later cofactor can be generated from MnII-2-NrdF, but only in the presence of O_2 _and NrdI protein [54,69]. RNR is responsible for the *de novo *conversion of ribonucleoside diphosphates into deoxyribonucleoside diphosphates and it is essential for DNA synthesis and repair [70]. The active site residues of RNR, in the template structure of NrdI protein (PDB 3N3A), include Ser8, Ser9, Ser11, Ser48, Asn13, Asn83, Thr14, Tyr49, Ala89 and Gly91, all of which are involved in a hydrogen bond network with the cofactor flavin mononucleotide isoalloxazine ring (FMN, PDB 3N3A) [71]. Interestingly, two of these residues, Ser8 and Tyr49, were predicted to make hydrogen bonds with all 10 selected ligands (Table 7**)**. The interaction between the top scoring compound **ZINC01585114 **(5-nitro-3, 4-diphenyl-2-furamide) and the residues from the predicted target cavities are shown in Additional file 4.

Furthermore, the drug-like molecule **ZINC00510419 **(3,4-bis (5-methylisoxazole-3-carbonyl)-1,2,5-oxadiazole 2-oxide was among the top ten selected molecules for three of the pathogen target proteins, showing good H-bond interactions. It ranked first against the targets Cp1002_0742 (MolDock score = -151.376, no. of H-bonds = 7) and Cp1002_1648 (MolDock score = -167.633, no. of H-bonds = 3) and ranked fourth against the target Cp1002_1676 (MolDock score = -154.064, no. of H-bonds = 4).

## Essential host homologous as putative targets

To compare the predicted EH protein targets to their host homologs, two approaches were taken. First, ClustalX (v2.1, http://www.clustal.org), a multiple sequence alignment program, was used to find different residues between bacterial and host proteins. As expected, a high percentage of residues was found to be conserved, but significant differences were also observed. Most percentage identities are between 35 and 50 (Table 8), except for fumarate hydratase, which shows 54% sequence identity to human and equine homologous proteins, but no hits in bovine and ovine proteomes.

**Table 8:** Percentage of sequence identity between *C*. pseudotuberculosis and host homologous proteins.

Protein Locus tag	Official full name	Percentage of Sequence Identity^#^			
		
		HS*	EC*	BT*	OA*
Cp1002_0385 **Adk**	Adenylate kinase	38	36	35	35

Cp1002_0692 **GapA**	Glyceraldehyde-3-phosphate dehydrogenase A	39	40	41	41

Cp1002_0728 **GlyA**	Serine hydroxymethyltransferase	43	45	45	45

Cp1002_0738 **FumC**	Fumaratehydratase class II	54	54	No Hits	No Hits

Cp1002_1005 **Gnd**	6-phosphogluconate dehydrogenase	48	48	48	48

Cp1002_1042 **AspA**	Aspartate ammonia-lyase	39	39	39	39

Next, to determine if the observed differences could be exploited in rational design of ligands selective to bacterial proteins, we focused on the predicted druggable cavities. A structural alignment to the host homologous proteins was performed and the cavities were compared in PyMol. In most cases, the DogSiteScorer predicted more than one cavity for each input Cp protein structure. The number of residues in the bacterial predicted cavity that differ from the residues in the cavity of the host protein, for all druggable pockets, varied from zero to seven (Table 9).

**Table 9:** Comparison of the residues from druggable cavities in *C. pseudotuberculosis* proteins and the corresponding residues in structurally aligned host protein cavities.

Protein Loci	Bacterial Residues for the Most Druggable Cavity Predicted by DGSS Server^#^	HS*	EC*	BT*	OA*
**Cp1002_0692** (Glyceralderayde 3-phosphate dehydrogenase)	Lys157	Asp35	Asp33	Asp33	Asp33
	
	Val174	Thr52	Thr50	Thr50	Thr50
	
	Arg229	Thr103	Thr101	Thr101	Thr101
	
	Asn311	Ala183	Ala181	Ala181	Ala181

**Cp1002_0385** (Adenylate kinase)	Phe35	Leu50	Leu52	Leu52	Leu43
	
	Ile53	Met68	Met70	Met70	Met61
	
	Thr64	Val79	Val81	Val81	Val72

**Cp1002_0728** (Serine hydroxymethyltransferase)	Cys70	Ala88	Thr86	Thr86	Thr86
	
	Ala99	Ser121	Ser119	Ser119	Ser119
	
	Ala101	Ser123	Ser121	Ser121	Ser121
	
	Trp177	Thr204	Thr202	Thr202	Thr202
	
	Pro361	Ala397	Ala395	Ala395	Ala395

**Cp1002_1005** (6-phosphogluconate dehydrogenase)	Ser55	Thr35	Thr161	Thr35	Thr35
	
	Met94	Leu74	Leu200	Leu74	Leu74
	
	Gln96	Lys76	Lys202	Lys76	Lys76
	
	Val104	Phe84	Phe210	Phe84	Phe84
	
	Ile148	Val128	Val254	Val128	Val128
	
	Gln268	Lys248	Lys374	Lys248	Lys248
	
	Pro269	His249	Tyr375	His249	His249

**Cp1002_1042** **(Aspartate ammonia-lyase)**	Gln193	His235	His257	His235	His235
	
	Ile428	Lys470	Lys492	Lys470	Lys470
	
	His447	Leu489	Leu511	Leu489	Leu489

^#^Drug score ≥ 0.80

*HS = Homo sapiens, EC = Equus caballus, BT = Bos taurus, OA = Ovis aries

For conserved host-homologous targets Cp1002_0385 (adk, Adenylate kinase), Cp1002_0692 (gapA, Glyceraldehyde 3-phosphate dehydrogenase), Cp1002_0728 (glyA, Serine hydroxymethyltransferase), Cp1002_0738 (fumC, Fumarate hydratase class II/fumarase), Cp1002_1005 (gnd, 6-Phosphogluconate dehydrogenase) and Cp1002_1042 (aspA, Aspartate ammonia-lyase/aspartase), three, four, five, zero, seven and three different residues were observed, respectively. Then, a more detailed analysis was performed for the predicted highest druggable cavity for each protein. The results are described below, together with information about the biological importance of each target protein.

**Cp1002_0692 **(**GapA, **Glyceraldehyde 3-phosphate dehydrogenase, GAPDH/G3PDH, EC 1.2.1.12) catalyzes the sixth step of glycolysis. In addition, GAPDH has recently been shown to be involved in several non-metabolic processes, including transcription activation, initiation of apoptosis [72] fast axonal or axoplasmic transport and endoplasmic reticulum to Golgi vesicle shuttling [73,74]. This enzyme has been reported as an anti-trypanosomatid and anti-leishmania drug target in structure-based drug design efforts [21-23]. Furthermore, it has been shown as an interesting putative drug and vaccine target in malaria pathogenesis [75]. Comparison of protein cavities reveals significant differences between bacterial and host proteins, with replacement of bacterial Lys157, Arg229 and Asn311 by Asp, Thr and Ala, respectively. Such differences result in a more basic cavity in bacteria, making it possible to rationally design selective ligands, especially negatively charged molecules, which interact with Lys157 and Arg229, or compounds able to form hydrogen bond to Asn311 (Additional file 5a).

Nucleoside monophosphate kinases vitally participate in sustaining the intracellular nucleotide pools in all living organisms. **Cp1002_0385 **(**Adk, **Adenylate kinase, EC 2.7.4.3) is a ubiquitous enzyme, which catalyzes the reversible Mg2^+^-dependent transfer of the terminal phosphate group from ATP to AMP, releasing two molecules of ADP [76]. Only one highly druggable cavity was predicted for adenylate kinase, with a druggability score = 0.81. Three residues in the bacteria cavity were different from the hosts: Leu, Met and Val in the hosts replaced Phe35, Ile53 and Thr64, respectively (Additional file 5b). These differences impact the cavity volume, since aromatic and bulky Phe is replaced by Leu, and the ability to make hydrogen bonds, through the replacement of a Thr by a Val. Therefore; the bacterial cavity is smaller and more hydrophilic, making it possible to envision rational design of selective ligands that interact with Thr64.

**Cp1002_0728 (GlyA**, Serine hydroxymethyltransferase EC 2.1.2.1) is an enzyme that plays an important role in cellular one-carbon pathways by catalyzing the reversible, simultaneous conversions of L-serine to glycine (retro-aldol cleavage) and tetrahydrofolate to 5,10-methylenetetrahydrofolate [77]. In Plasmodium, serine hydroxymethyltransferase (SHMT) has been reported as an attractive drug target [78]. For this protein 3 residues were observed different between bacteria and host: Ala99 and Ala101 replaced two Ser residues while Trp177 replaced Thr (Additional file 5c). At first glance these changes could have a big impact in the active site, generating a considerably more hydrophilic pocket in the hosts. However, careful inspection of the pocket reveals that the side chains of these residues are not turned towards the pocket, in such a way that these differences probably would not allow rational design of selective ligands.

**Cp1002_0738 **(**FumC, **Fumaratehydratase class II/fumarase EC 4.2.1.2) catalyzes the reversible hydration/dehydration of fumarate to S-malate during the ubiquitous Krebs cycle, through the aci-carboxylate intermediate subsequent to olefin production [79]. There are two classes of fumarases; Class I fumarases, composed of heat-labile, iron-sulfur (4Fe-4S) homodimeric enzymes, only found in prokaryotes; and Class II fumarases, made of thermostable homotetrameric enzymes [80] found in both prokaryotic and eukaryotic mitochondria. Class II belongs to a superfamily that also includes aspartate-ammonia lyases, arginino-succinatases, d-crystallins and 3-carboxy-cis, cis-muconate lactonizing enzymes. All these enzymes release fumarate from different substrates, ranging from adenylosuccinate to malate [81-84]. FumC of *Escherichia coli *is the first member of class II fumarases family whose structure has been solved and provided most of the structural information [85]. Inhibition of fumarase in the tricarboxylic acid cycle (TCA) has been reported as a potential molecular target of bismuth drugs in *Helicobacter pylori *[86]. Comparison of the active site cavity of this protein, which is formed in the interface of three monomers, revealed no differences between bacteria and hosts (additional file 5d).

**Cp1002_1005 **(**Gnd, **6-Phosphogluconate dehydrogenase EC 1.1.1.44) is an enzyme from the pentose phosphate pathway. It forms ribulose 5-phosphate from 6-phosphogluconate. The enzyme 6-phosphogluconate dehydrogenase is a potential drug target for the parasitic protozoan *Trypanosoma brucei*, the causative organism of human African trypanosomiasis [87]. Three druggable sites with score > 0.80 were detected in this protein. As opposed to the observation for other proteins, the most druggable predicted cavity (score = 0.88) was not the active site. Leu, Lys and Val residues in the hosts replace residues Met94, Gln96 and Ile148 in the bacterial cavity, respectively (Additional file 5e). The most significant of these differences is the replacement of Gln by Lys, which could make binding of negative molecules more favorable to the host proteins.

**Cp1002_1042 **(**AspA, **Aspartate ammonia-lyase/aspartase EC 4.3.1.1) catalyzes the deamination of aspartic acid to form fumarate and ammonia [88]. Recent progresses to prepare enantiopure l-aspartic acid derivatives, highly valuable tools for biological research and chiral building blocks for pharmaceuticals and food additives, make it a target of interest for industrial applications. On the other hand, the important role that it plays in microbial nitrogen metabolism makes it a putative drug target in overcoming bacterial pathogenesis [89]. Based on the sequence alignment for this protein, two significant differences in residues are observed in the most druggable pocket: bacterial His447 and Ile428 are replaced by Leu and Lys in host proteins. Such differences should allow rational ligand design. It is interesting to note that additional differences in the position of helices that contain these residues increase the difference between the active sites (Additional file 5f).

Based on the above-mentioned analyses, we conclude that it would be difficult to rationally design selective ligands for **Cp1002_0738 **(**FumC, **Fumaratehydratase class II), since no residue differences were observed in the most druggable cavity, and for **Cp1002_0728 (GlyA**, Serine hydroxymethyltransferase), where the side chains of differing residues are not turned toward the druggable pocket. On the other hand, for putative essential and homologous targets that include **Cp1002_0692 **(**GapA, **Glyceraldehyde 3-phosphate dehydrogenase), **Cp1002_0385 **(**Adk, **Adenylate kinase), **Cp1002_1005 **(**Gnd, **6-Phosphogluconate dehydrogenase) and **Cp1002_1042 **(**AspA, **Aspartate ammonia-lyase), significant differences were observed in druggable pockets, suggesting that despite the existence of a host homologous protein they could be good targets for the design of ligands, selective only to the bacterial proteins.

## Conclusion

Here, for the first time, the genomic information was used to determine the conserved predicted proteome of 15 strains of *C. pseudotuberculosis*, along with their three-dimensional structural information. Even though the structural information discussed is fully computationally predicted, and could therefore deviate from eventually solved experimental structures, we have been careful to concentrate on the analysis of protein models for which there were good templates which provided high quality models, minimizing this concern. The data presented here can effectively contribute in guiding further research for antibiotics and vaccines development. The final dataset can provide valuable information in designing molecular biology and immunization experiments in animal models for validating the targets of a pathogen, as well as in experimental structure determination protocols.

The criterion for target selection in *C. pseudotuberculosis* was stringent, resulting in a small set of prioritized putative drug and vaccine targets, of which four are essential and non-homologous and six are essential and host homologous proteins. For the latter, a detailed structural comparison between the residues of the predicted cavities of host and pathogen proteins has been performed, showing in most cases the potential for the development of selective ligands. Therefore, we suggest that the whole set can be considered for antimicrobial chemotherapy, especially the four essential non-host homologous targets.

The *in silico *approaches followed in this study might aid in the development of novel therapeutic drugs and vaccines in a broad-spectrum of hosts at intraspecies level against *C. pseudotuberculosis*. Furthermore, the strategy described here could also be applied to other pathogenic microorganisms.

## Authors' contributions

Coordinated entire work: SSH RSF VA DB. Performed all *in silico *analyses: SSH RSF ST SBJ NBS FDP LCG. Cross-analyzed genome contents, pan-modelome construction, conserved pan-modelome, subtractive modelome approach, virtual screening & docking analyses and residue level structural comparison: SSH RSF ST FDP AI SCS SA DB AGT. Provided timely consultation and reviewed the manuscript: VA AI SCS SA DB NBS LCG AA AM AS VACA AGT. Read and approved the final manuscript: RSF SSH ST AI SCS SBJ SA DB NBS LCG AGTAA AM AS VA. Conceived and designed the work: SSH RSF VA DB. Analyzed the data: SSH RSF ST AI SCS SBJ SA DB NBS LCG AA AB LJ AGTAM AS VA. Wrote the paper: SSH RSF ST.

## Conflict of interest

The authors declare that they have no competing interests.

## Supplementary Material

Additional file 1 Docking representation of the best drug-like compound **ZINC75109074 **in the most druggable protein cavity of **Cp1002_0515 **(**MtrA**, DNA-binding response regulator). Three hydrogen bonds were observed with Thr73, Asp48 and Arg116. Click here for file

Additional file 2 Docking representation of compound **ZINC00510419 **in the most druggable protein cavity of **Cp1002_0742 **(**IspH**, 4-hydroxy-3-methyl but-2-enyl diphosphate reductase). Residues Cys39, Thr225, Ser250, His68 and Asn252 are predicted to make seven hydrogen bonds to this ligand. Click here for file

Additional file 3 Docking representation of the best drug-like compound **ZINC00510419 **in the most druggable protein cavity of **Cp1002_1648 **(**TcsR, **Two component transcriptional regulator). Hydrogen bonds were observed with residues Val76, Gln185 and Asn193. Click here for file

Additional file 4 Docking representation of the best drug-like compound **ZINC04721321 **in the most druggable protein cavity of **Cp1002_1676 **(**NrdI **protein). Hydrogen bonds were observed with residues Ser8, Thr13 and Leu116. Click here for file

Additional file 5 (a-f) Comparison among the most druggable cavities from essential bacterial and the respective host homologue proteins. Protein structures are shown as cartoon (green for the bacterial protein and gray for *Ovis aries *host protein). Other host proteins are not shown for simplicity, but the same substitutions were present in all host proteins analyzed. Residues that differ in the bacterial and host cavity are highlighted in sticks and labeled (bacterial labels in green and host labels in black). a) **Cp1002_0692 **(Glyceralderayde 3-phosphate dehydrogenase); b) **Cp1002_0385 **(adenylate kinase); c) **Cp1002_0728 **(serine hydroxymethyltransferase); d) **Cp1002_0738 **(fumarate hydratase class II) the site shown is formed by three monomers, which are represented in green, blue and orange. No residues are highlighted, since the active sites are identical between bacteria and host; e) **Cp1002_1005 **(6-phosphogluconate dehydrogenase); f) **Cp1002_1042 **(aspartate ammonia-lyase). Figures were prepared with the PyMol. Click here for file
