# Supplementary figures and images for: Proteome scale comparative modeling for conserved drug and vaccine targets identification in Corynebacterium pseudotuberculosis
Source: BMC Genomics. 2014 Oct 27;15(Suppl 7):S3. doi: 10.1186/1471-2164-15-S7-S3 (PMC4243142; doi:10.1186/1471-2164-15-S7-S3)

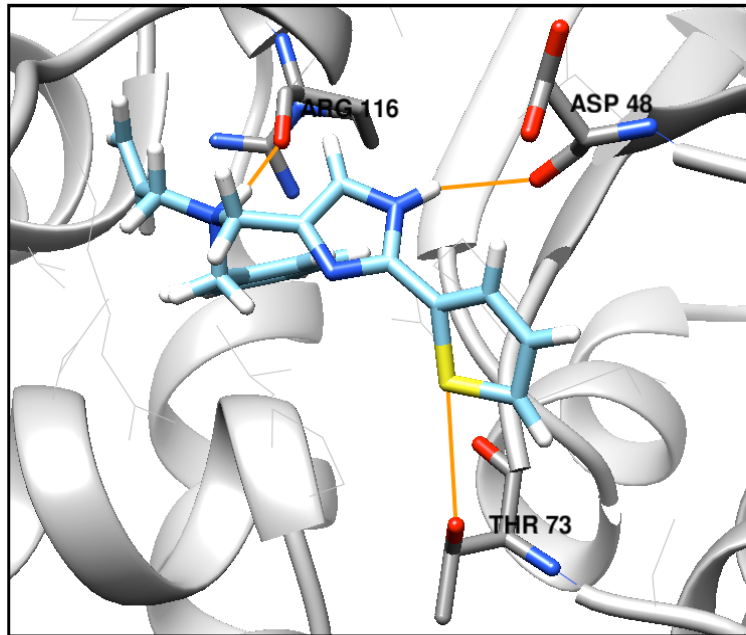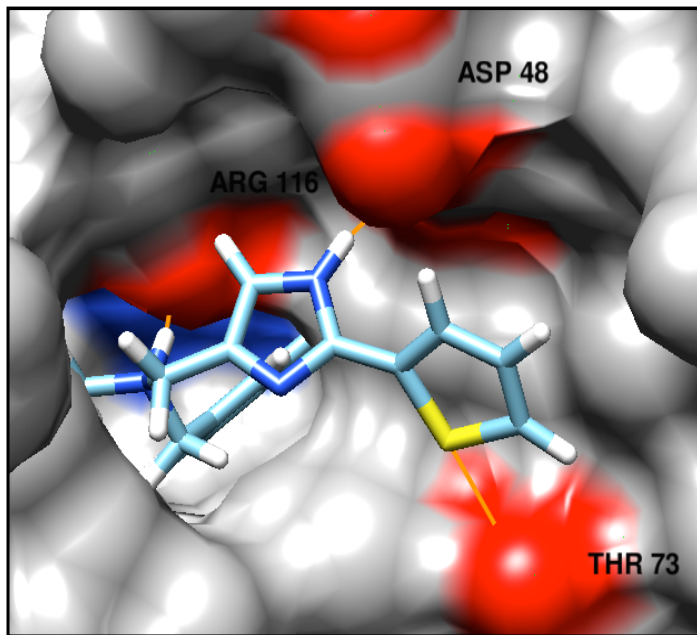

Supplement: Additional file 1 — Docking representation of the best drug-like compound ZINC75109074 in the most druggable protein cavity of Cp1002_0515 (MtrA, DNA-binding response regulator). Three hydrogen bonds were observed with Thr73, Asp48 and Arg116. [file 1471-2164-15-S7-S3-S1.pdf]

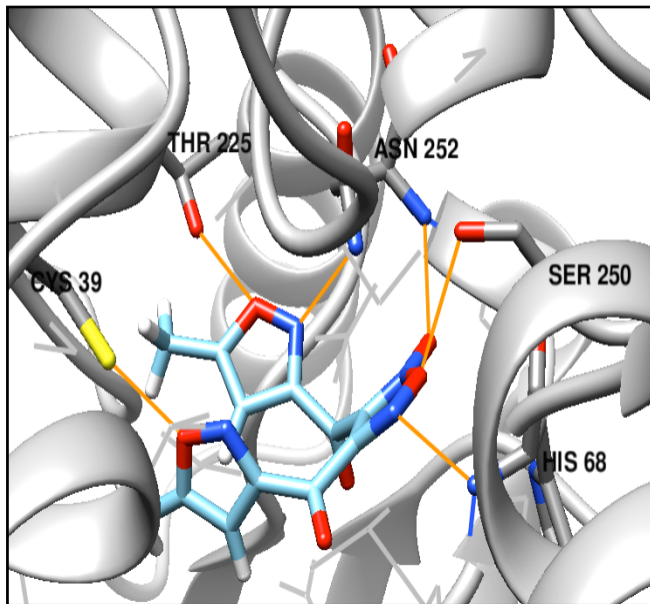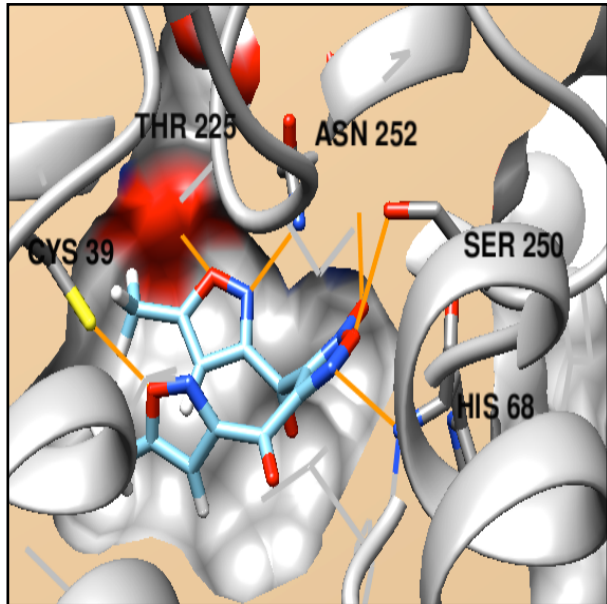

Supplement: Additional file 2 — Docking representation of compound ZINC00510419 in the most druggable protein cavity of Cp1002_0742 (IspH, 4-hydroxy-3-methyl but-2-enyl diphosphate reductase). Residues Cys39, Thr225, Ser250, His68 and Asn252 are predicted to make seven hydrogen bonds to this ligand. [file 1471-2164-15-S7-S3-S2.pdf]

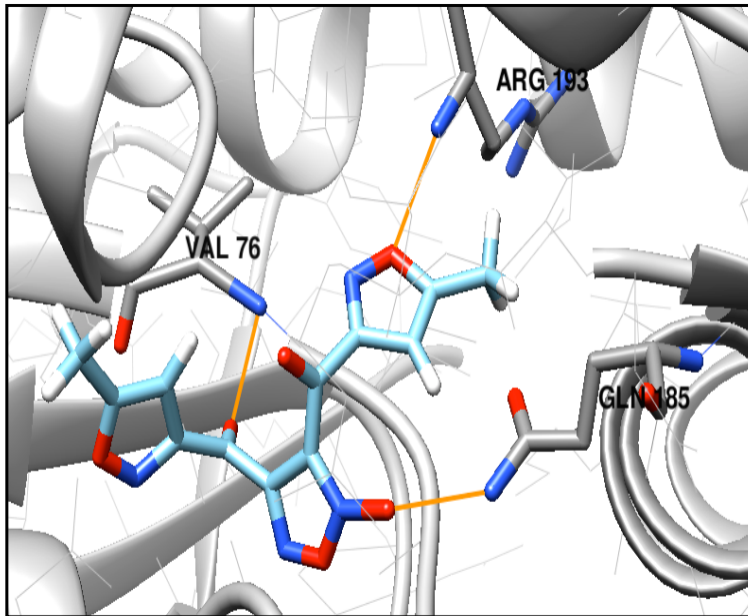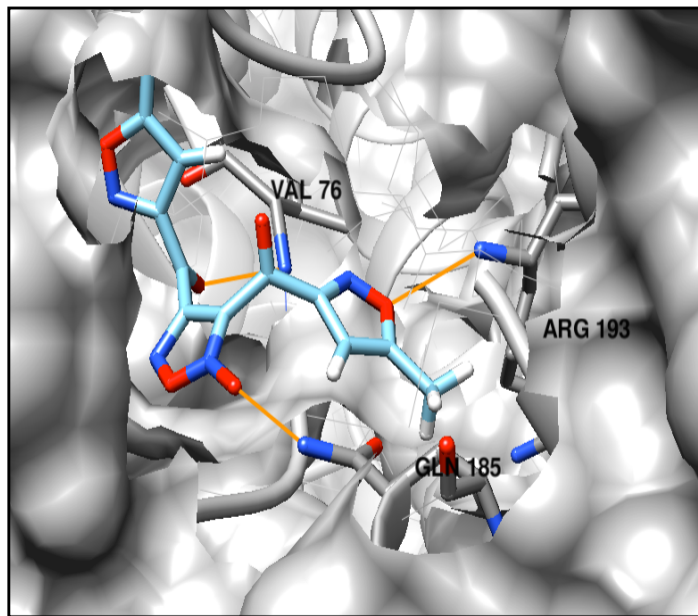

Supplement: Additional file 3 — Docking representation of the best drug-like compound ZINC00510419 in the most druggable protein cavity of Cp1002_1648 (TcsR, Two component transcriptional regulator). Hydrogen bonds were observed with residues Val76, Gln185 and Asn193. [file 1471-2164-15-S7-S3-S3.pdf]

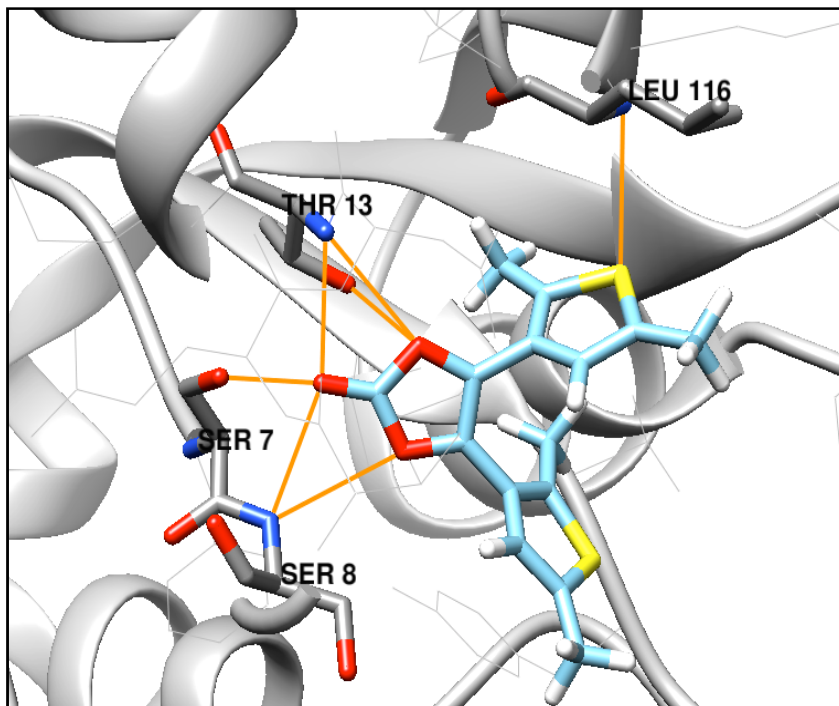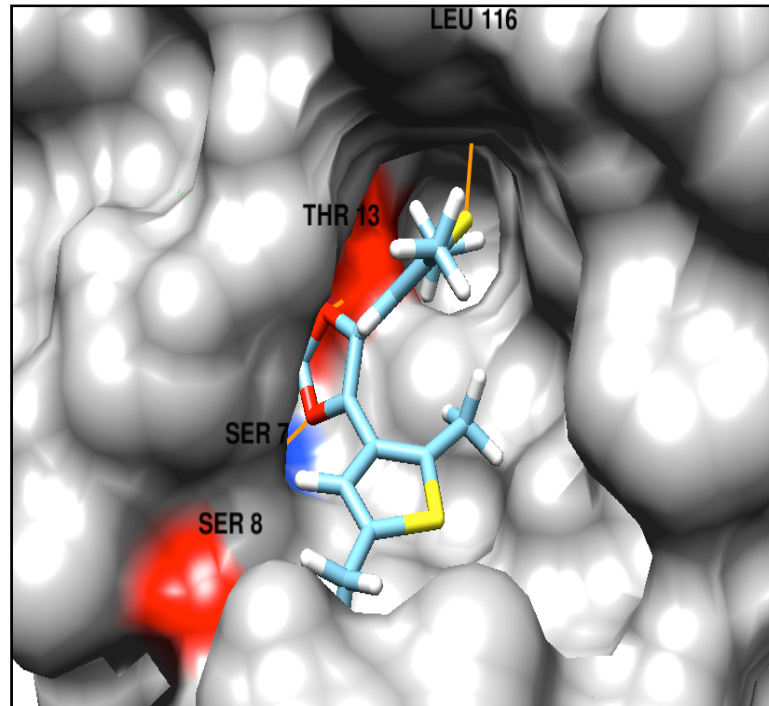

Supplement: Additional file 4 — Docking representation of the best drug-like compound ZINC04721321 in the most druggable protein cavity of Cp1002_1676 (NrdI protein). Hydrogen bonds were observed with residues Ser8, Thr13 and Leu116. [file 1471-2164-15-S7-S3-S4.pdf]

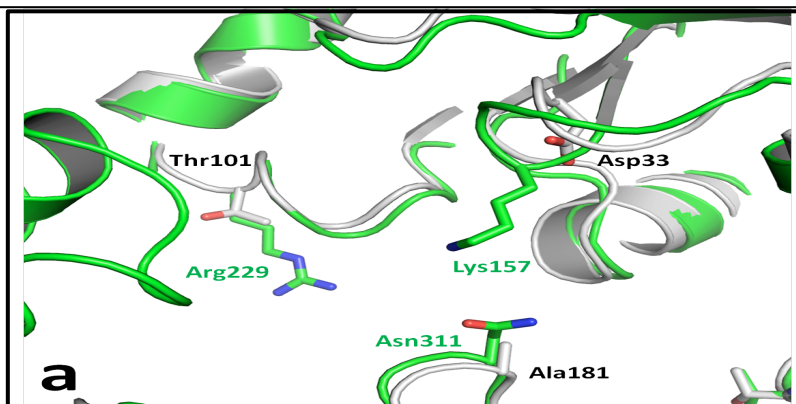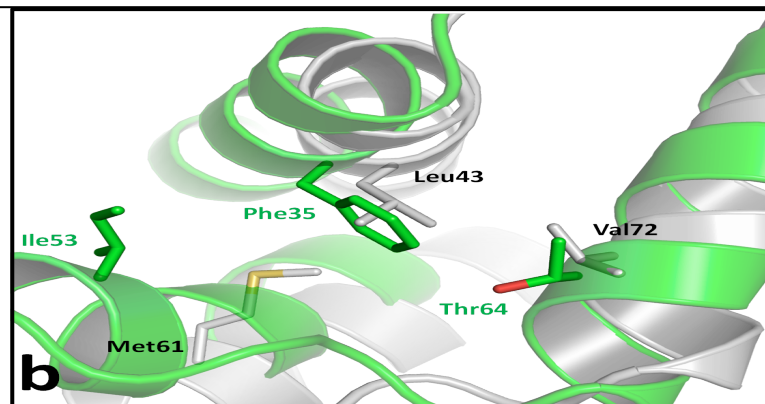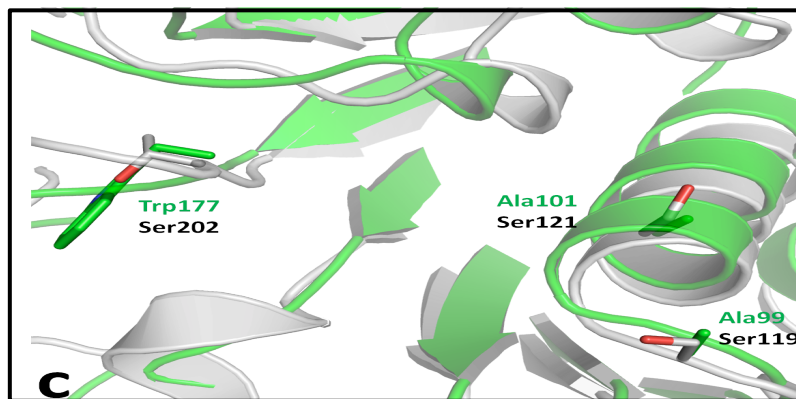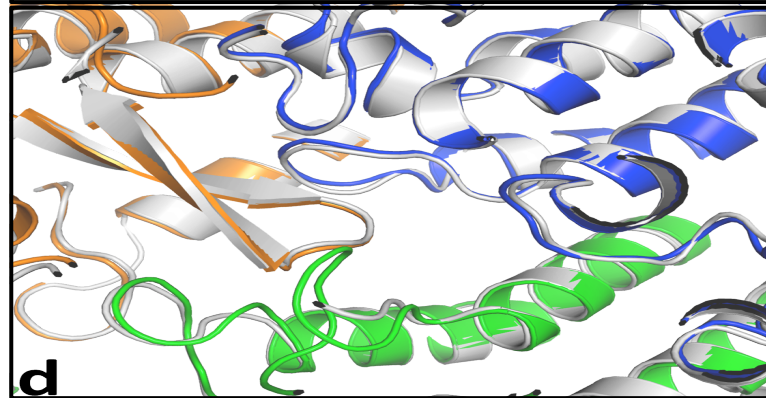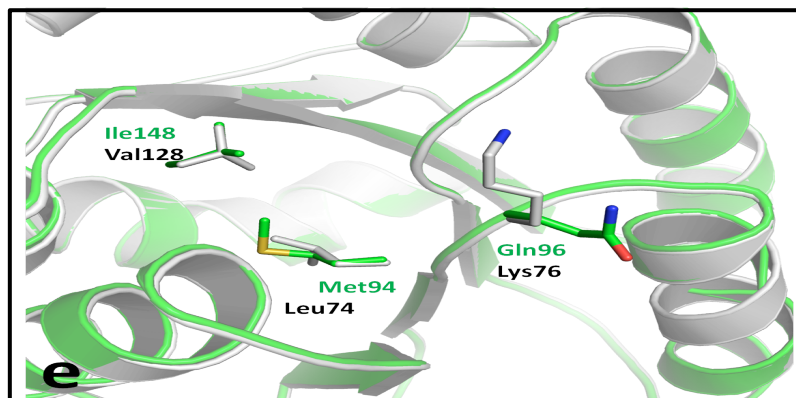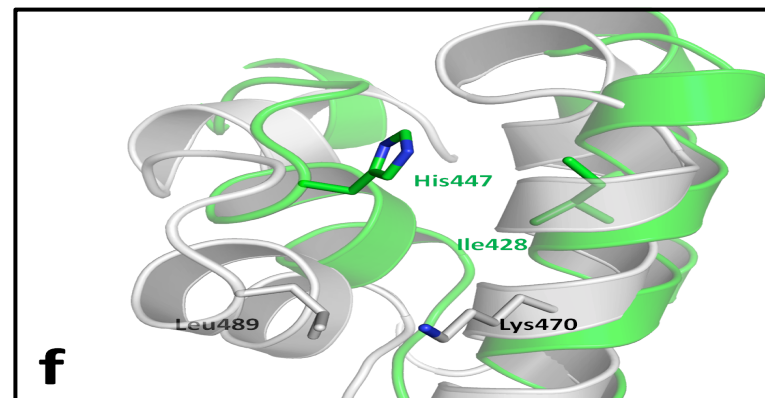

Supplement: Additional file 5 (a-f) — Comparison among the most druggable cavities from essential bacterial and the respective host homologue proteins. Protein structures are shown as cartoon (green for the bacterial protein and gray for Ovis aries host protein). Other host proteins are not shown for simplicity, but the same substitutions were present in all host proteins analyzed. Residues that differ in the bacterial and host cavity are highlighted in sticks and labeled (bacterial labels in green and host labels in black). a) Cp1002_0692 (Glyceralderayde 3-phosphate dehydrogenase); b) Cp1002_0385 (adenylate kinase); c) Cp1002_0728 (serine hydroxymethyltransferase); d) Cp1002_0738 (fumarate hydratase class II) the site shown is formed by three monomers, which are represented in green, blue and orange. No residues are highlighted, since the active sites are identical between bacteria and host; e) Cp1002_1005 (6-phosphogluconate dehydrogenase); f) Cp1002_1042 (aspartate ammonia-lyase). Figures were prepared with the PyMol. [file 1471-2164-15-S7-S3-S5.pdf]
